# Synchronous presentation of acute cholecystitis and acute appendicitis successful treatment in one step laparoscopic procedure. A case series and literature review

**DOI:** 10.1016/j.ijscr.2021.106296

**Published:** 2021-08-10

**Authors:** Seyed Mohammad Nahidi, Khuram Khan, Christopher Engler, Sharang Tickoo, Carina Biggs

**Affiliations:** a Department of Surgery, Wyckoff Heights Medical Center, Brooklyn, 11237, NY, USA; b St George's University, Grenada, West Indies, Grenada

**Keywords:** Emphysematous cholecystitis, Necrotizing appendicitis, Four ports, Case series

## Abstract

**Introduction:**

Hundreds of thousands of cholecystectomies and appendectomies are performed in the United States annually. Due to the prevalence of cholecystitis and appendicitis, a subset of patients will require both operations. The limited literature describing these patients supports a laparoscopic approach over open surgery; consistent with the advantages of laparotomy over open surgery in the treatment of each condition individually.

**Case presentation:**

We report two cases where a patient presented with cholecystitis and appendicitis simultaneously. An abdominal computer tomography (CT) scan revealed the presence of the two diagnoses, which was then confirmed by an abdominal ultrasound. A four-access port was utilized for simultaneous appendectomy and cholecystectomy.

**Discussion:**

A review of the literature indicates that simultaneous infection with appendicitis and cholecystitis is rare, and thus clinical presentation, lab work, and imaging studies are all needed to support such a diagnosis. Potential findings on imaging in these patients may include distended gallbladder with thickened wall and fluid-filled dilated appendix with mural enhancement. In the event that both clinical presentation and further work-up indicate both pathologies, laparoscopic intervention is suitable. A four-access port is deemed the conservative approach to dealing with such cases.

**Conclusion:**

Finding a single diagnosis responsible for a patient's illness is a high priority in an acute care setting, a concept known as diagnostic parsimony. However, it is inevitable that very common illnesses will be comorbid in a subset of patients, and physicians should be prepared to consider contemporaneous illness in the isolated circumstances it is warranted.

## Highlights

• Simultaneous appendicitis and cholecystitis is rare.
• Computer tomography is an accurate diagnostic tool in cases such as this.
• A four-access port is deemed the conservative approach to dealing with such cases.

## 1 Introduction

Acute appendicitis is one of the most common causes of acute abdominal pain and frequently warrants emergent abdominal surgery worldwide [1]. Another cause of acute abdominal pain is acute cholecystitis which typically develops in patients with symptomatic cholelithiasis. Acute appendicitis and acute cholecystitis are among the most common pathologies seen in general surgery practice, however, simultaneous appendicitis and cholecystitis in a single patient have only been rarely reported previously. Having awareness of the possibility of this double diagnosis will allow clinicians to entertain this differential in patients who present with acute abdomen where the physical examination and imaging studies suggest a mixed picture. A successful initial diagnosis in these cases is essential in preventing the need for a subsequent operation for the second cause of their acute abdominal pathology as well as reducing the risk of mortality from a second undiagnosed infection. We also aim to describe the use of standard laparoscopic cholecystectomy port placements to achieve both cholecystectomy and appendectomy in a single setting. This case series has been reported in line with the PROCESS Guideline [2].

## 2 Case presentation 1

A 68-year-old male patient, with a past medical history of hypertension and chronic obstructive pulmonary disease and denied any past surgical history presented to our institution complaining of diffuse abdominal pain over the past 4 days associated with constipation, nausea, and vomiting. He described the pain as crampy, diffuse pain rated as 8/10. He mentioned that the pain was more prominent in the epigastric and the right upper quadrant. The patient denied any episodes of this nature in the past. He claimed that his last bowel movement was four days ago which he described as non-bloody. The patient tested negative for Covid-19. The patient reported a distended abdomen, along with nausea and vomiting. A nasogastric tube was placed and 400 cc bilious fluid was suctioned. On the physical exam, his abdomen was distended and diffusely tender to palpation in all quadrants with rebound tenderness. Rest of the examination was within normal limits.

Upon admission, the patient's vital signs were tachycardic, 123 bpm, and hypertensive, 142/106 mmHg. His Hemoglobin was 12.1 g/dL, and hematocrit was 36.4%. The patient's arterial blood gas presented with a pH of 7.45, pCO2 of 27 mmHg, pO2 of 62 mmHg, and HCO3 of 18.8 mEq/L. He also had an increase in BUN, creatinine, and lactic acid; 67 mg/dL, 2.20 mg/dL, and 3.4 mmol/L.

**Fig. 1:**
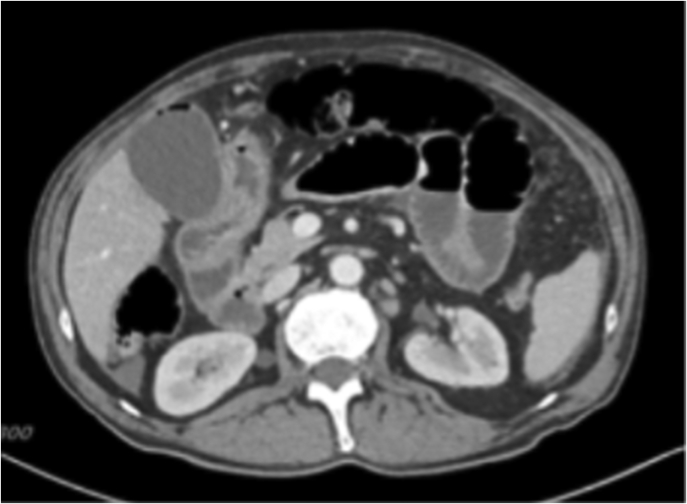
A transverse CT scan of the abdomen identifying severe enteritis and biliary gas within the gallbladder and the bile duct.

Computer tomography (CT) of the abdomen and pelvis (Fig. 1) was performed. Severe enteritis throughout the jejunum and ileum, biliary gas within the gallbladder and bile duct, and diverticulosis within the sigmoid colon were identified.

**Fig. 2:**
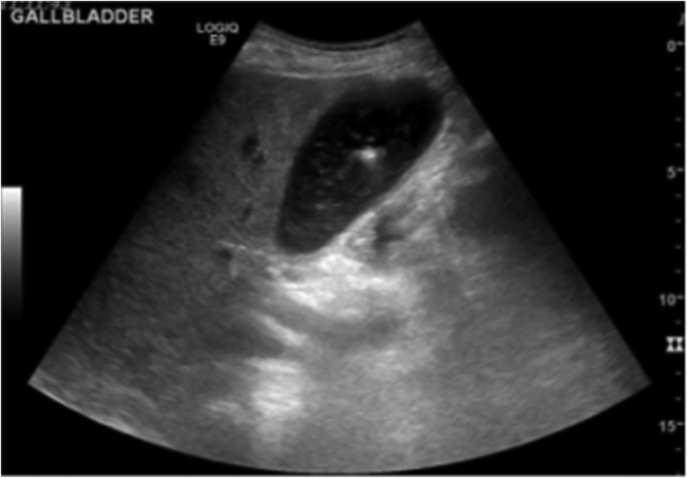
Ultrasound of the gallbladder identified thickening of the gallbladder and a small stone.

Ultrasound identified enlarged heterogeneous, and hyperechoic liver. His gallbladder (Fig. 2) was distended and contained debris in the small stones. The gallbladder wall was thickened and adjacent infiltration was present. Acute cholecystitis was suspected. Surgery was consulted and after the clinical and radiographic exam, the patient was scheduled for urgent laparoscopic cholecystectomy. Prior to surgery, the patient was placed on prophylactic antibiotics; Ciprofloxacin and Metronidazole.

Entry into the abdomen was performed using the open Hasson technique. Upon access, purulent peritonitis was encountered. The gallbladder was identified and appeared to be frankly necrotic and a subtotal cholecystectomy was done as extensive inflammation of the critical view was noted. During laparoscopic irrigation of the abdomen, a pinpoint perforation was identified in the small bowel in the left lower quadrant. After the discovery of the perforation, the laparoscopy was converted to a midline laparotomy. The small bowel was inspected from the ligament of Treitz to terminal ileum and diffuse patches of ischemia were noted on the jejunum, without areas of infarct. A pinpoint perforation was discovered on the mid jejunum. The appendix was also identified and appeared to be necrotic and perforated which prompted an appendectomy. Five centimeters of the small bowel was resected and left in discontinuity. The abdomen was left open and covered with an Abthera wound vacuum the patient then remained intubated and was transferred to the intensive care unit (ICU). The postoperative course was significant for pH 7.11, lactate acid was 1.1 mg/dL, and urine output 15-30 mL/h. The patient's tachycardia resolved. CT angiography was done overnight to rule out any vascular infarct and there was no evidence seen on CT angiography. A bedside echocardiogram was performed and results were normal without evidence of fluid overload or hypokinesia. The pathological evaluation confirmed cholecystitis and gangrenous appendicitis.

The patient was taken back to the operative room on postoperative day three. A small bowel perforation was identified at the distal small bowel which had been previously left in discontinuity. Another resection was performed and again the small bowel was left in discontinuity. An abdominal washout was performed due to continued sepsis and the Abthera vacuum was replaced and the patient was taken back to ICU. The patient was scheduled for another return to the operating room (OR) with abdominal washout and exploration the following day. A similar procedure was done. Abthera wound vacuum was replaced and the patient was transferred to the ICU. The patient remained unstable with significant lactic acidosis and leukocytosis after postoperative day 1. Unfortunately, the patient suffered a cardiac arrest on postoperative day 6.

## 3 Case presentation 2

A 41-year-old man presented to the emergency department complaining of right upper abdominal pain of 10 days duration. The patient endorsed associated symptoms of vomiting, fever, and chills. On physical examination, the abdomen was soft with mild tenderness on the right upper and lower quadrants however there was no rebound tenderness or guarding present.

Laboratory analysis of the patient's blood revealed no leukocytosis. An abdominal CT scan revealed a fluid-filled dilated appendix with mural enhancement concerning acute appendicitis as well as diffuse gallbladder wall edema with no biliary duct dilation however peri-portal free fluid was present. These findings were confirmed by the use of an abdomen ultrasound and the concern for acute appendicitis and acute cholecystitis became apparent.

The patient underwent an urgent laparoscopic cholecystectomy and appendectomy. The operations were performed sequentially and the port site placement was as follows: an umbilical Hasson port insertion and three-port insertions in the epigastric region and right midclavicular and anterior axillary subcostal spaces as for standard laparoscopic cholecystectomy. The gallbladder was visualized and was acutely inflamed with marked distention and an edematous wall. The appendix was also visualized and appeared dilated and hyperemic. After the gallbladder was removed, the appendectomy was performed using the same cholecystectomy ports, modifying instruments placement as follows: the epigastric and midclavicular ports were used as working ports to remove the appendix. Pathological evaluation of the appendix revealed changes consistent with acute appendicitis and gallbladder pathology showed acute cholecystitis superimposed on chronic cholecystitis with focal gangrenous change. The patient tolerated the procedure well with no complications and was discharged on postoperative day 3 and was seen in the office for follow up after 2 weeks and was doing well without any complaints.

## 4 Discussion

In an acute care setting, an immediate goal is generally to find a single diagnosis suitable for the constellation of symptoms. However, in the setting of management of chronic conditions, comorbidities - even unrelated ones - are hardly surprising, and patients will frequently necessitate management of contemporaneous illnesses. Similarly, common acute illnesses have the potential to manifest contemporaneously. For example, the lifetime risk of acute appendicitis is 8.6% in male patients and 6.7% in females. While lifetime risk is difficult to quantify, the annual risk of acute cholecystitis is 0.63% before 50 years of age and 2.1% above 50 [3], [4]. In the United States, approximately 500,000 cholecystectomies and 300,000 appendectomies are performed annually [5]. In spite of the fact that reported cases of appendicitis and cholecystitis together are rare [6], [7], [8], [9], it is crucial that the co-occurrence remains a possible differential diagnosis due to the prevalence and severity of both diseases. These comorbid patients typically present with severe abdominal pain with features consistent with both illnesses, such as foci of pain in the upper right quadrant as in cholecystitis or the hallmark periumbilical to lower-right localization of pain that typifies the clinical presentation of appendicitis.

In many of these patients, an abdominal CT scan reveals inflammatory or edematous changes in both the gallbladder and appendix [6], [8], [10]. A CT scan of the abdomen has become the standard of care in cases of severe lower abdominal pain due to its advantages in being more accurate than ultrasound in diagnosing non-gallstone pathologies [11]. Particularly in cases where appendicitis and cholecystitis are occurring at the same time, it is crucial to obtain a CT scan of the abdomen since ultrasound can cause misinterpretation of results due to anatomical variations in appendix positioning [11]. CT Scans of the abdomen were found to reduce the number of false negatives for appendicitis from 24% to 3% which has led to quicker management from decreased misdiagnosis which lessens further complications such as a perforated appendix. In terms of cholecystitis CT scans and sonograms were found to have comparable accuracy [11]. As approximately 8% of gallstones are isodense to surrounding bile, ultrasonography is still the preferred choice for isolated right upper quadrant abdominal pain [9]. However, there are instances such as in our first case where initial imaging only revealed changes consistent with cholecystitis and not appendicitis, yet the appendicitis was later elucidated via exploratory laparoscopy. However, in our second case imaging was a crucial aspect of the appendicitis diagnosis as the clinical signs that typically elicit pain in appendicitis were negative.

Emphysematous cholecystitis is a rare and severe form of acute cholecystitis. In many cases of emphysematous cholecystitis, studies have shown a 15-20% mortality, which may often be linked to the incidence of gallbladder gangrene and perforation [12], [13]. Similar to a patient in a previous report, our patient also exemplified a friable gallbladder due to gangrenous changes, which were further discussed to be due to gall bladder distention and inflammation, leading to ischemic changes and necrosis. The incidence of these gangrenous changes ranged from 2%-30% of all patients with acute cholecystitis, and incidence further increases with other factors (i.e. age >50) [14]. Hemostatic abnormalities and hypoperfusion from sepsis serve as a potential mechanistic explanation for the necrosis throughout the patient's small bowel and perforated gallbladder [15], [16]. A possible takeaway is that physicians treating severe appendicitis in emergency settings should maintain elevated clinical suspicion for sepsis-related injury and subsequent infection elsewhere in the gut.

While the first step calls for accurate diagnosis of this rare clinical scenario the second important element is appropriate management to prevent complications such as peritonitis and possibly death. Although successful treatment of appendicitis and cholecystitis without surgical intervention has been reported [9], this cannot ensure either condition will not reoccur later on. In one of the presented cases, the patient underwent a single operation to remove both the appendix and the gallbladder. While case reports exist of open cholecystectomy and appendectomy, the majority of reported cases favor a laparoscopic approach - indeed, in one of the cases presented, we see a worse outcome in the patient in which this was not an option. [7] The most common approach, granting adequate access to both right-upper and right-lower quadrants, involves five access ports: a larger (11 - 12 mm) access port immediately superior to the umbilicus and four smaller (5 mm) ports, three in the right upper quadrant and one in the left lower quadrant. [8], [14], [16] While they performed this approach in the management of their case, Victory et al. hypothesized a more conservative approach involving only four ports by omitting the 5 mm left lower quadrant port and positioning the central supraumbilical port offset to the left. As the goal of laparoscopy is to minimize surgery-related morbidity, the possibility of an equally effective but more conservative approach warrants further investigation. The circumstances of emergent surgery may necessitate either or both appendectomy and cholecystectomy be performed as open procedures; however, both procedures have previously been shown to have poorer outcomes than their laparoscopic counterparts and should be avoided when possible.

Overall, while finding a single consistent diagnosis is a priority in the management of acute conditions, there should still remain strong clinical suspicion of multiple simultaneous illnesses in the rare but demonstrable event that a single diagnosis does not fit the presentation of the patient's illness. Though there may be a common etiological cause in these cases, a common etiology is not necessary to explain the comorbidity of very common illnesses - specifically, the sheer volume of appendicitis and cholecystitis cases in the United States is sufficient enough that a small number of contemporaneous cases should be expected annually [16]. Additionally, initial pathologies may provoke subsequent conditions as the patient's condition deteriorates. This underlying concept known as Hickam's Dictum applies, which states that diagnostic parsimony is a guideline and not a rule. Due to the rarity of these cases, individual care teams will need to exercise clinical judgment in the pursuit of optimal patient care. However, there is some indication that a four or five-port laparoscopy is the best currently available approach [6], [7].

## 5 Conclusion

The instance of a dual diagnosis of acute appendicitis and acute cholecystitis presented simultaneously brings it to the attention of clinicians who frequently evaluate patients for acute abdomen. The two-pronged risk of perforation necessitates prompt diagnosis and surgical intervention. We are able to describe laparoscopic cholecystectomy and appendectomy through the utilization of the same ports used for standard laparoscopic cholecystectomies. By describing the readjustment of instrument placement for better visualization of the appendix, physicians can know how to quickly handle this rare emergent situation through the use of port placement well known to general surgeons due to the high incidence of laparoscopic cholecystectomies performed annually.

## Sources of funding

There are no fundings provided for this case report.

## Ethical approval

N/A.

## Consent

“Written informed consent was obtained from the patient for publication of this case report and accompanying images. A copy of the written consent is available for review by the Editor-in-Chief of this journal on request.”

## Registration of research studies

N/A.

## Guarantor

Khuram Khan, MD.

## Provenance and peer review

Not commissioned, externally peer-reviewed.

## CRediT authorship contribution statement

Seyed Mohammad Nahidi - Write-up.

Sharang Tickoo - Write-up.

Khuram Khan - Resident Surgeon/Write-up.

Christopher Engler - Resident Surgeon/Write-up.

Carina Biggs - Attending Surgeon.

## Declaration of competing interest

There are no conflicts of interest in this case report.
